# Eating disorders, body image and media exposure among adolescent girls in rural Burkina Faso

**DOI:** 10.1111/tmi.13340

**Published:** 2020-01-26

**Authors:** Valentin Terhoeven, Christoph Nikendei, Till Bärnighausen, Mamadou Bountogo, Hans‐Christoph Friederich, Lucienne Ouermi, Ali Sié, Guy Harling

**Affiliations:** ^1^ Centre for Psychosocial Medicine University Hospital Heidelberg Heidelberg Germany; ^2^ Institute for Global Health University College London London UK; ^3^ Heidelberg Institute of Global Health (HIGH) University of Heidelberg Heidelberg Germany; ^4^ Department of Global Health and Population Harvard T.H. Chan School of Public Health Boston MA USA; ^5^ Africa Health Research Institute (AHRI) Somkhele and Durban South Africa; ^6^ Centre de Recherche en Santé de Nouna (CRSN) Nouna Burkina Faso; ^7^ Department of Epidemiology & Harvard Center for Population and Development Studies Harvard T.H. Chan School of Public Health Boston MA USA

**Keywords:** anorexia nervosa, bulimia nervosa, binge eating disorder, body dissatisfaction, West Africa, anorexie mentale, boulimie nerveuse, frénésie alimentaire, insatisfaction corporelle, Afrique de l'Ouest

## Abstract

**Objective:**

Body dissatisfaction and eating disorders (ED) among young females may increase in limited‐resource settings as exposure to media and higher‐resource cultures increases. We examined ED prevalence and its predictors among adolescent girls in rural north‐western Burkina Faso.

**Methods:**

Fieldworkers interviewed 696 female adolescents aged 12‐20 years in the Nouna Health and Demographic Surveillance System (HDSS). ED were evaluated using the Structured Clinical Interview for DSM‐5 (SCID‐5), self‐perceived appearance and body ideal were measured using Thompson and Gray’s Contour Drawing Rating Scale (CDRS) and eating disorder predictors by the Eating Disorder Examination Questionnaire (EDE‐Q). We assessed media exposure to magazines, radio, television, and the internet.

**Results:**

16% of respondents had a BMI below WHO age‐standardised 5th percentile, while 4% were above the 85th percentile; most respondents wanted to be larger. DSM‐5 criteria for anorexia nervosa (AN) were fulfilled by four of 696 respondents (0.6%), those for bulimia nervosa by none, and those for binge eating disorder by two (0.3%). In multivariable regression, more AN symptoms were associated with greater EDE‐Q body dissatisfaction, desiring a thinner body and a history of sexual harassment or assault, but not with media exposure. A thinner desired body was associated with greater media exposure, higher BMI z‐score and greater EDE‐Q disordered eating.

**Conclusion:**

ED were very rare in rural Burkinabé female adolescents, but factors predictive of ED in higher‐resource settings were also predictive of ED precursor symptoms here. Our findings suggest that increasing media exposure in resource‐limited settings may lead to increased body dissatisfaction, and potentially to increased future ED prevalence.

## Abstract (fr)

**Objectif:**

L'insatisfaction corporelle et les troubles de l'alimentation (TA) chez les jeunes femmes peuvent augmenter dans des régions à ressources limitées, à mesure qu'augmente l'exposition aux médias et aux cultures plus nanties. Nous avons examiné la prévalence des TA et leurs prédicteurs chez les adolescentes dans les zones rurales du nord‐ouest du Burkina Faso.

**Méthodes:**

Les enquêteurs de terrain ont interrogé 696 adolescentes âgées de 12 à 20 ans dans le Système de Surveillance Démographique et de Santé (SSDS) de Nouna. Les TA ont été évalués à l’aide de l’Interview Clinique Structurée du DSM‐5 (SCID‐5), l’apparence perçue de soi et l’idéal corporel ont été mesurés à l’aide de l’échelle de *Thompson et Gray Contour Drawing Rating* (CDRS) et les prédicteurs des TA par le questionnaire d’Examen des Troubles de l’Alimentation (EDE‐Q). Nous avons évalué l'exposition aux médias par les magazines, la radio, la télévision et Internet.

**Résultats:**

16% des répondants avaient un IMC inférieur au 5^è^ percentile normalisé selon l'âge de l'OMS, tandis que 4% dépassaient le 85^è^ percentile. La plupart des répondants souhaitaient être plus grosses. Les critères du DSM‐5 pour l'anorexie mentale (AM) étaient remplis par 4 des 696 répondants (0,6%), ceux de la boulimie mentale par aucun et ceux de la frénésie alimentaire par 2 (0,3%) répondants. Dans la régression multivariée, des symptômes accrus d’AM étaient associés à une plus grande EDE‐Q insatisfaction corporelle, au désir d'un corps plus mince et à des antécédents de harcèlement sexuel ou d'agression sexuelle, mais non à une exposition médiatique. Inversement, le souhait d’un corps plus mince était associé à une plus grande exposition aux médias, à un score z de l’IMC plus élevé et à un EDE‐Q de TA plus élevé.

**Conclusion:**

Les TA étaient très rares chez les adolescentes burkinabé en zone rurale, mais les facteurs prédictifs des TA dans les pays à ressources élevées étaient également prédictifs dans cette région. Nos résultats suggèrent qu'une exposition accrue aux médias dans des régions à ressources limitées pourrait conduire à une insatisfaction corporelle accrue et potentiellement à une prévalence future accrue des TA.

## Introduction

According to the Diagnostic and Statistical Manual of Mental Disorders, 5th edition (DSM‐5) 1, anorexia nervosa (AN), bulimia nervosa (BN) and binge eating disorder (BED) are mental disorders characterised by abnormal eating habits (e.g. extreme dietary restraint or uncontrollable eating), compensatory behaviours (e.g. self‐induced vomiting) and overvalued ideas about weight and shape (e.g. fear of body weight increase) that severely affect physical as well as mental health 2. AN is characterised by extremely low body weight accompanied by the fear of becoming fat; BN comprises repeated episodes of uncontrollable eating (i.e. binge eating) followed by compensatory behaviours (e.g. vomiting); BED is defined as binge eating like BN but without compensatory behaviours, leading to severe overweight. There is evidence that eating disorders lead to serious medical comorbidities and complications 3, as well as psychosocial burden; 97% of those with eating disorders (ED) also have at least one comorbid psychosocial diagnosis, mainly unipolar depression and anxiety disorders 4. Moreover, ED are associated with the highest mortality rate of all mental disorders 5.

ED occur most frequently in young females in higher income countries (HIC) 6; however, there is evidence that these disorders also occur in other groups 7. The inclusion of ED in the Global Burden of Disease Study in 2013 contributed to their greater recognition in the global health community 8, 9. It has been suggested that ED will increase in historically non‐high‐risk populations such as those living in lower‐ and middle‐income countries (LMICs), due to ongoing industrialisation, urbanisation and globalisation 10. There is some research evidence for ED in Africa, including a report of two AN cases and one BN case in urban Cameroon based on DSM‐IV 11 and a study describing a therapeutic programme for 10 anorexic and 21 bulimic Tunisian teenagers 12.

One risk factor contributing to the development of ED in HICs is media exposure, particularly in adolescents, who are more vulnerable to messages imparted via mass media that create an unrealistic body image 13, 14, 15. It is likely that media exposure is also an important risk factor in LMICs. Indeed, a Tanzanian study found that increased media exposure (television and internet access), and exposure to HIC culture, predicted ED symptoms 16. One likely underlying mechanism for media affecting ED symptoms in LMICs is changes in body image (i.e. self‐perception of body and body ideal) due to the adoption of HIC beauty ideals. Such a mechanism is supported by a review showing that girls and women living in sub‐Saharan Africa (SSA) preferred a larger body compared to African girls and women who had emigrated to HIC 17, 18. The authors of this review hypothesised that media exposure might disseminate HIC beauty ideals in SSA causing changes in body image 18, so that young African girls and women move from a larger towards a thinner ideal body image. Such a change may lead to greater body dissatisfaction, which, in turn, is recognised as a key factor for the development and maintenance of ED 19, 20, 21.

The epidemiology of ED in Africa is still in its infancy. A recent review underlined the importance of studies assessing eating pathology according to formal psychiatric diagnostic systems (e.g. DSM‐5) 22. In epidemiological studies, no cases of AN have yet been reported in SSA using the DSM‐IV criteria, but the new, less stringent, DSM‐5 criteria may mean that more African women now fulfil AN criteria 22. This is in line with warnings that ED such as AN exist in SSA (as seen in case studies) and their relevance in this context should not be underestimated 23. Point prevalence of BN in young African women has been estimated to be 0.87%^22^, which is within the range reported for young women in HIC 6, 24 and Latin America 25. The diagnosis of BED is acknowledged as a distinct eating disorder for the first time in DSM‐5 and we are unaware of any estimates of BED in SSA.

We, therefore, assessed the DSM‐5 ED criteria for AN, BN and BED in a rural West African adolescent female population in this exploratory study. We first hypothesised that a few Burkinabé adolescent girls may be at risk of ED or even fulfil DSM‐5 ED criteria. Second, we investigated how body mass index (BMI) was related to self‐perception of body (current body vs. body ideal), hypothesising that, in line with literature on adolescents in SSA, study participants would demonstrate body dissatisfaction 26 and misperceive their weight 27. Third, we investigated media access, hypothesising that, given estimates that 95% of rural Burkinabé adolescents live in severe poverty 28, media exposure would be low, but might still predict potential ED risk factors such as body dissatisfaction.

## Methods

### Study design

We used baseline data from a cohort of adolescents aged 12‐20 in rural Burkina Faso, which is part of the ARISE Adolescent Health Study. The cohort was drawn from the Nouna Health and Demographic Surveillance System (HDSS) overseen by the Centre de Recherche en Santé de Nouna (CRSN). The HDSS covers a population of over 100,000 residents and comprises the town of Nouna and 59 surrounding villages in the Boucle du Mouhoun Province in western Burkina Faso 29. The cohort was sampled using a two‐stage stratified sampling procedure. First, 10 Nouna HDSS villages were purposively sampled to ensure all five main local ethnicities were included. Then, a sample of 1795 adolescents who were age‐eligible on 1 October 2017 was drawn based on the 2015 HDSS census, respecting the ethnic composition of all age‐eligible HDSS adolescents. Second, we drew a simple random sample of 749 age‐eligible adolescents from one of the seven sectors of Nouna town. This sample thus represented the ethnic and rural‐urban balance of the HDSS as a whole, though not the national or regional balance of these factors. For this study, we restricted our sample to female respondents.

### Procedures

Baseline interviews were conducted in November and December 2017 at respondents’ homes, with interviews conducted in either French or a local language. The study collected self‐reported information on socio‐demographics, behaviours, health practices and health outcomes, using tablet computers. Approvals for this study were obtained from the Institutional Ethics Committee of the CRSN, village elders, participants (written consent/assent) and parents/guardians (written consent if participant aged <18). The Ethics Commission of the Medical Faculty of the University of Heidelberg exempted the study, because only anonymised data were provided to non‐CRSN staff.

### Measures

The Structured Clinical Interview for DSM‐5 (SCID‐5) is a semi‐structured expert interview guide of the American Psychiatric Association (APA) used to determine DSM‐5 diagnoses 20. We used the DSM‐5 chapter ‘Feeding and Eating Disorders’ to evaluate current and lifetime diagnoses of AN, BN and BED. We also created SCID sum scores for each diagnosis based on fulfilled DSM‐5 criteria, with a maximum possible value of three for AN and five for BN and BED. We also used the Eating Disorder Examination Questionnaire (EDE‐Q), a self‐report scale which assesses attitudes and behaviours related to abnormal eating habits 30. We used the shortened 7‐item French version of the EDE‐Q, which covers (i) dietary restraint, (ii) shape/weight overvaluation, and (iii) body dissatisfaction 31. This version of the EDE‐Q has been previously validated 32. The EDE‐Q uses a 7‐point Likert scale with a mean score of ≥4 as a cut‐off for clinical ED psychopathology relevance 33. We conducted a factor analysis of the responses to the EDE‐Q questions to confirm that the three factors of the questionnaire were indeed present in this sample. We then summed response values for variables within the three confirmed factors to generate a score for each EDE‐Q factor.

Weight and height were measured twice each by the fieldworkers; BMI was calculated based on the mean of each pair of measures; four respondents were excluded for infeasible weight or height values. We categorised weight using both the CDC BMI‐for‐age growth charts and WHO charts, which adapt the CDC charts for worldwide use 34. The former allows comparison with HIC studies, while the latter is more appropriate for our population. For the CDC charts, we used the 5th and 85th percentiles as bounds for normal weight; for WHO charts, we used −2 and + 1 standard deviations for these bounds. In regression analyses, we used the WHO BMI‐for‐age z‐scores. Although evidence on SSA adolescent nutritional status and BMI scores is not currently available, it appears that human growth is similar worldwide in healthy, well‐nourished individuals absent environmental or socioeconomic constraints 35. Furthermore, the three most‐used classification systems for adolescent weight substantially agree with one another 36. We use the WHO 2007 references in this study given past recommendations for their use in SSA, to facilitate comparability and time trend‐analyses of overweight and obesity rates within the continent 37, and worldwide 38.

We assessed body image with Thompson and Gray’s Contour Drawing Rating Scale (CDRS) 39. The CDRS consists of nine female body contour images sorted from underweight (1‐3) through normal weight (4–6) to overweight (7–9). Participants were asked to indicate, first, which image best fits how they think their body currently looks (current body self‐perception) and, second, which image best fits how they wish to look like (ideal body image). We calculated body dissatisfaction by subtracting current body self‐perception from ideal body image (i.e. negative values represent a desire to be smaller, positive values to be bigger). The CDRS has good validity and test–retest reliability in Australian early‐adolescent girls 40. The scale has not been validated in SSA; however, it uses figural line drawings without ethnicity‐specific facial or body features, and thus should be broadly applicable 41.

We measured aggregate media exposure by summing frequency of use across four media. The frequencies of use of three media (magazines, television, and the internet) were scaled as 0 for ‘never’, 1 for ‘some hours per month’, 2 for ‘some hours per week’, and 3 for ‘some hours a day’. The frequency of use of one medium (radio) was 0 for ‘never’, 1 for ‘<1 h a day’, 2 for ‘1–2 h a day’; 3 for ‘≥2 h a day’). This scaling resulted in a maximum possible value of aggregate media exposure of 12 16. Finally, since sexual harassment and violence may affect body image and psychological well‐being, we created a four‐point scale based on lifetime experience of verbal harassment, unwanted sexual touching, attempted rape, and rape.

### Statistical analysis

After generating descriptive statistics for all relevant variables, we ran bivariate Pearson correlations between each of the three EDE‐Q factor scores (i.e. dietary restraint, shape/weight overvaluation, body dissatisfaction) and both CDRS difference score and aggregate media exposure. We conducted multivariable Poisson regression to investigate whether known ED predictors in HICs also predicted our participants’ SCID AN, BN, and BED sum scores. These predictors included aggregate media exposure (continuous), current school enrolment (binary), experience of sexual violence, age (12–13, 14–15, 16–17, and 18–20 years) and the three EDE‐Q factor scores. We adjusted the regressions for village of residence.

We then ran multivariable linear regressions to evaluate how potential ED risk factors were associated with (i) the CDRS difference score between ‘self‐perceived current body’ and ‘ideal body’ and (ii) the EDE‐Q body dissatisfaction sum score. Bivariate analyses were conducted for all five outcomes and explanatory variables in a preliminary analysis. Data were analysed using SPSS (version 25; SPSS Inc., Chicago, IL., USA).

### Ethics

All procedures contributing to this work comply with the ethical standards of the relevant national and institutional committees on human experimentation and with the Helsinki Declaration of 1975, as revised in 2008.

## Results

**Figure 1:**
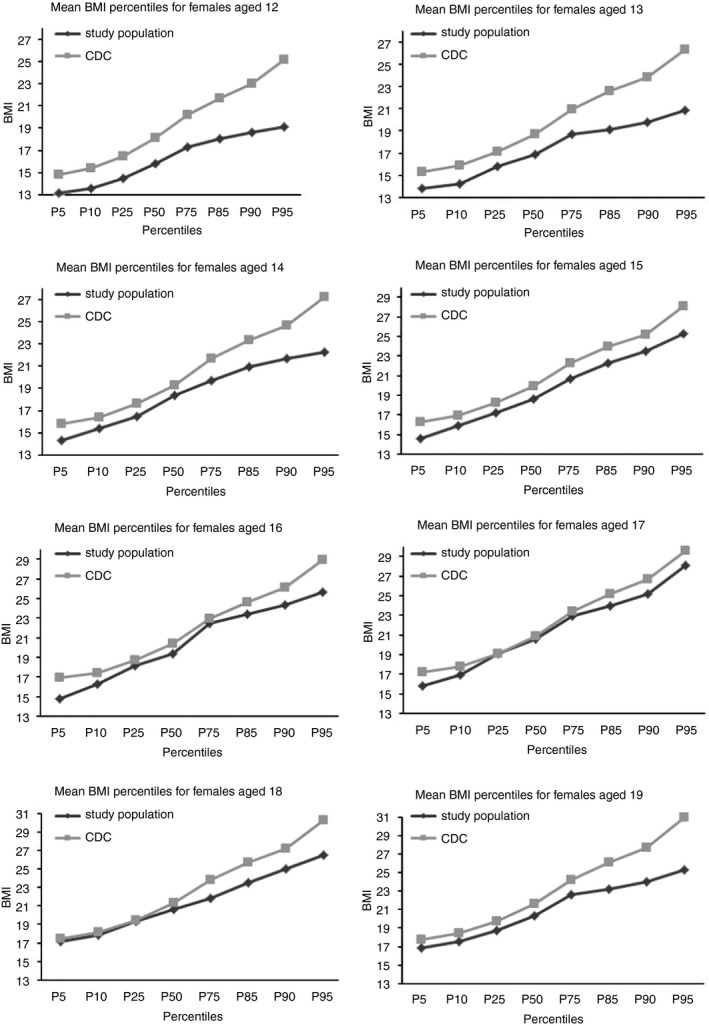
Comparison of BMI percentiles by age to the CDC BMI‐for‐age Growth Charts for female adolescents aged 12 to 20.

A total of 696 of 1276 adolescent girls sampled (54.5%) were located and consented to participate. Non‐response was overwhelmingly due to migration (30.2% of non‐participants) or non‐contact (61.1%); only 13 individuals and 7 responsible adults refused consent to participate. Respondent characteristics are shown in Tables 1 and 2. BMI ranged from 11.8 to 38.2 kg/m^2^ (mean = 18.8, standard deviation [SD] = 3.2) and mean values by age were slightly below CDC growth chart levels (Figure 1): while an expected number were normoweight, 16.1% were underweight and only 4.2% were overweight. Applying WHO growth charts gave a similar proportion of normoweight respondents, but fewer underweight and more overweight respondents.

**Table 1:** Descriptive statistics for continuous study variables (*n* = 696)

	Possible range	*n* missing	Mean (SD)	Median (IQR)
CDRS				
Self‐perceived current body	1 to 9	21	4.37 (2.24)	4 (3 to 6)
Desired ideal body	1 to 9	19	6.26 (2.06)	7 (5 to 8)
CDRS difference score	−8 to 8	29	1.89 (2.55)	2 (0 to 4)
Aggregate media exposure score	0 to 12		2.20 (1.55)	2 (1 to 3)
SCID				
AN sum score	0 to 3	30	0.41 (0.03)	0 (0 to 1)
BN sum score	0 to 5	6	0.03 (0.01)	0 (0 to 0)
BED sum score	0 to 5	6	0.05 (0.01)	0 (0 to 0)
EDE‐Q				
Total score	0 to 49	15	5.20 (5.92)	4 (0 to 8)
Dietary restraint	0 to 21		0.34 (0.04)	0 (0 to 0)
Shape/weight overvaluation	0 to 14	12	2.95 (0.06)	0 (0 to 4)
Body dissatisfaction	0 to 14	9	3.47 (0.07)	0 (0 to 4)
WHO BMI‐for‐age z‐scores		8	−0.70 (0.04)	−0.6 (−1.4 to 0.1)
Experience of sexual violence	0 to 4	11	0.50 (0.96)	0 (0 to 1)

AN, Anorexia Nervosa; BED, binge eating disorder; BMI, body mass index; BN, Bulimia Nervosa; CDRS, Thompson and Gray’s Contour Drawing Rating Scale; EDE‐Q, Eating Disorder Questionnaire; IQR, interquartile range; SCID, structured clinical interview for DSM‐5; SD, standard deviation; WHO, World Health Organization.

**Table 2:** Descriptive statistics for categorical study variables

	*n*	%
Resident in Nouna town	380	29.8
Currently in school	381	54.7
Age (years)		
12–13	357	28.0
14–15	321	25.2
16–17	321	25.2
18–20	275	21.6
BMI (WHO scale)		
Underweight‐for‐age (<−2SD)	83	12.0
Normal weight‐for‐age	564	82.0
Overweight‐for‐age (>+1SD)	41	6.0
BMI (CDC scale)		
Underweight‐for‐age (<5th percentile)	111	16.1
Normal weight‐for‐age	549	79.7
Overweight‐for‐age (>85th percentile)	29	4.2

BMI, body mass index; CDC, Centers for Disease Control and Prevention; IQR, interquartile range; SD, standard deviation; WHO, World Health Organization.

Twenty participants met all three AN criteria, of whom four (0.61%, 95% confidence interval [CI]: 0.02 to 1.55%) were additionally classified as underweight according to CDC growth charts and thus fulfilled the DSM‐5 criteria for AN. For BN, no‐one met *criterion C* (frequency of compensatory behaviours ≥ once per week) or *criterion D* (figure and weight strongly affect self‐esteem) and thus BN prevalence was zero (95% CI: 0.00 to 0.53%). For BED, two respondents met all four criteria (0.30%, 95% CI: 0.04 to 1.04%). EDE‐Q scores were higher for the shape/weight overvaluation and body dissatisfaction factors than for dietary restraint (Table 1). Factor analysis revealed mediocre sampling adequacy (Kaiser–Meyer–Olkin = 0.609), but three factors accounting for 83.6% of the variance had eigenvalues>1 and loaded as expected (Table S1) 42.

**Figure 2:**
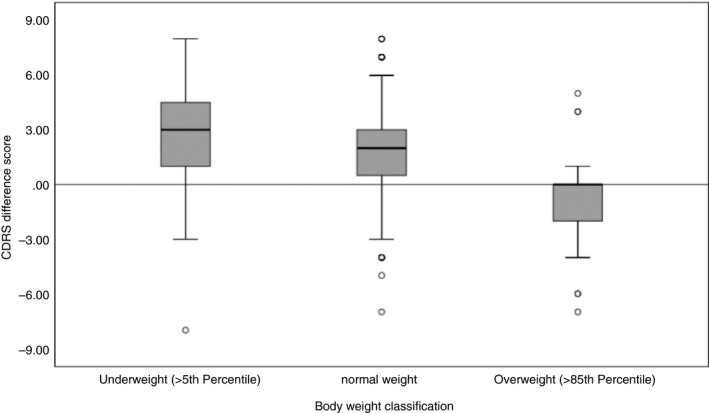
Difference between ‘self‐perceived current body’ and ‘ideal body’. Based on Thompson and Gray’s Contour Drawing Rating Scale (CDRS) according to body weight classification. Positive scores indicate the desire for a bigger body, negative scores indicate the desire for a thinner body, and zero scores indicate satisfaction with body silhouette.

On average, respondents stated that their ideal body was larger than their current self‐perceived body, based on the CDRS (Table 1). Mean CDRS differences between current and ideal bodies were 3.1 (interquartile range [IQR] 1‐5) among those classified as underweight by CDC growth charts (*n* = 106), 1.8 (IQR 0‐3) among those classified as normoweight (*n* = 525) and −0.8 (IQR −2‐0) among those classified as overweight (*n* = 29) (Figure 2).

Among BMI *underweight‐for‐age* respondents, 64.2% self‐perceived their current body as underweight, 26.4% as normoweight and 9.4% as overweight; 78.3% desired a larger body, 16% were exactly satisfied, and 5.7% desired a thinner body. Among BMI *normal weight‐for‐age* respondents*,* 44.4% perceived their current body in the normal range, 36.2% as underweight, and 19.4% as overweight; 69.3% desired a larger body*,* while 19.4% were exactly satisfied, and 11.2% desired a thinner body. Of the 29 BMI *overweight‐for‐age* respondents, 18 self‐perceived themselves as overweight, four as underweight and seven as normoweight; six desired a larger body, ten were satisfied and 13 desired a thinner body.

The CDRS difference score was significantly correlated with the EDE‐Q sum scores for body dissatisfaction (*r*_(664)_ = 0.19, *P* <0.001) and dietary restraint (*r*_(667)_ = −0.19, *P* <0.001), but not for shape/weight overvaluation (*r_(_*_663)_ = −0.02, *P* = 0.69). Media exposure was not significantly correlated with the EDE‐Q total score, but was significantly associated with the CDRS difference score (*r*_(667)_ = −0.14, *P* <0.001).

### Predictors of ED symptoms and body dissatisfaction

Bivariate regressions for predictors of SCID sum scores are shown in Table S2. In multivariable regression, higher SCID AN scores were significantly associated with greater EDE‐Q body dissatisfaction and a history of sexual harassment/violence (Table 3). Higher SCID BN score were significantly associated with a history of sexual harassment. Higher SCID BED scores were significantly associated with greater EDE‐Q shape/weight overvaluation, a desire for a larger body (i.e. higher CDRS difference score), and higher BMI. None of the three scores were significantly associated with media exposure.

**Table 3:** Multivariable Poisson regression of SCID ED score values

	SCID AN sum score		SCID BN sum score		SCID BED sum score	
	IRR	95%CI	IRR	95%CI	IRR	95%CI
Media exposure	1.04	0.96–1.12	1.03	0.78–1.38	0.80	0.62–1.03
Currently not in vs in school	1.07	0.81–1.41	1.33	0.49–3.60	1.75	0.77–3.98
WHO BMI‐for‐age *z*‐score	0.97	0.86–1.10	1.47	0.93–2.34	1.45	1.02–2.06
Sexual harassment/violence	1.18	1.06–1.33	1.57	1.10–2.25	1.28	0.93–1.76
Age (years)						
12–13	0.69	0.45–1.05	1.22	0.27–5.46	0.39	0.11–1.38
14–15	1.02	0.69–1.43	0.85	0.21–3.40	0.54	0.19–1.55
16–17	1.05	0.71–1.45	1.78	0.56–5.73	1.42	0.62–3.26
18–20	1.00		1.00		1.00	
CDRS difference score	0.97	0.91–1.02	1.14	0.93–1.40	1.25	1.07–1.46
Resident in Nouna vs. elsewhere	1.20	0.92–1.58	1.53	0.58–4.06	1.13	0.50–2.55
EDE‐Q						
Dietary restraint	1.09	1.00–1.19	0.99	0.68–1.43	1.18	0.92–1.50
Shape/weight overvaluation	1.03	0.99–1.07	1.13	0.99–1.29	1.15	1.05–1.27
Body dissatisfaction	1.05	1.02–1.09	1.06	0.94–1.20	1.07	0.98–1.17

AN, Anorexia Nervosa; BED, binge eating disorder; BMI, body mass index; BN, Bulimia Nervosa; CDRS, Thompson and Gray’s Contour Drawing Rating Scale; CI, confidence interval; EDE‐Q, Eating Disorder Questionnaire; IRR, incidence rate ratio; SCID, structured clinical interview for DSM‐5; WHO, World Health Organization.

Bivariate linear regressions for predictors of CDRS difference score and EDE‐Q body dissatisfaction are shown in Table S3. In multivariable regression, higher CDRS difference scores were significantly associated with all three EDE‐Q factors, lower BMI, and greater media exposure. Higher EDE‐Q body dissatisfaction was significantly associated with greater EDE‐Q shape/weight overvaluation, dietary restraint, higher CDRS difference scores, and living in Nouna (Table 4).

**Table 4:** Multivariable linear regression of body dissatisfaction and body image

	CDRS difference score		EDE‐Q body dissatisfaction factor	
	B	95%CI	B	95%CI
Media exposure	−0.13	−0.24 to−0.01	0.07	−0.10 to 0.24
Currently not in vs in school	−0.29	−0.67 to 0.10	0.30	−0.25 to 0.86
WHO BMI‐for‐age *z*‐scores	−0.69	−0.86 to−0.52	−0.16	−0.41 to 0.10
Sexual harassment/violence	−0.17	−0.36 to 0.03	−0.11	−0.39 to 0.17
Age (categorised)	−0.03	−0.22 to 0.15	0.08	−0.18 to 0.34
CDRS difference score	−	−	0.27	0.16 to 0.38
Nouna vs. other residence	0.31	−0.09 to 0.71	0.66	0.008 to 1.23
EDE‐Q				
Dietary restraint	−0.31	−0.48 to−0.13	0.29	0.04 to 0.54
Shape/weight overvaluation	−0.08	−0.14 to−0.01	0.53	0.44 to 0.61
Body dissatisfaction	0.13	0.08 to 0.19	–	−

B, regression coefficient; BMI, Body Mass Index; CDRS, Thompson and Gray’s Contour Drawing Rating Scale; CI, Confidence Interval; EDE−Q, Eating Disorder Questionnaire; WHO, World Health Organization.

## Discussion

We assessed the prevalence of both DSM‐5 criteria eating disorder and psychological and media‐related risk factors for ED in a rural female West African adolescent sample. While only six of 696 respondents fulfilled all DSM‐5 criteria for AN, BN or BED, risk factors for ED (such as body image disturbance and body dissatisfaction) were more prevalent. The four individuals (point prevalence 0.6%) meeting all diagnostic criteria for AN may represent the first identification of AN cases based on the DSM‐5 diagnostic criteria in an SSA population‐based survey 43. Finding such cases is, nonetheless, in line with predictions that some African adolescents would probably screen positive for AN based on the new, laxer, DSM‐5 criteria 22.

None of our respondents fulfilled all the criteria for BN (95% CI 0–0.53%), which is below the already‐low point prevalence found in other, mostly urban, African studies. We also identified two respondents meeting all criteria for BED (point prevalence 0.29%); we believe that this is the first SSA reported prevalence of BED, reflecting BED having previously been included in ‘Eating Disorder Not Otherwise Specified’ (EDNOS) in DSM‐IV. African ENDOS prevalence has previously been reported to be 4.5% 22, but this included some DSM‐5 AN and BN cases not meeting the more stringent DSM‐IV criteria.

Compared to other studies in SSA, the BN and BED prevalences measured in our study are even lower. This comparison may reflect systematic differences in context: All previous ED studies in SSA were conducted in higher‐income communities (urban Egypt, urban Kenya, and urban and rural Tanzania) 16, 44, 45. The only other West African ED study, conducted in rural north‐eastern Ghana, assessed only AN and found no cases 46. Rural Burkina Faso’s extreme poverty may mean scarcer food availability compared to previously studied SSA populations, limiting the opportunities for binge eating – either with or without compensatory behaviours. This understanding is supported by the low levels of dietary restraint measured by the behavioural dimension of the EDE‐Q in our sample. ED‐related binge eating is normally associated with unwanted weight gain due to attempts to regulate undesired mood states. Most of our respondents showing BED‐related behaviours, however, wished to be larger rather than suffering from weight gain, suggesting that BED symptom reporting here probably reflects unrelated issues.

While clinical ED symptoms were rare in our study, our data point to possible increased ED prevalence in the future. Scores on the two cognitive/affective EDE‐Q dimensions were higher than for behavioural dimension, suggesting some respondents were overvaluing and dissatisfied with their bodies. This hypothesis is also supported by the 10 BMI‐underweight respondents (9.4% of all those underweight) who self‐perceived themselves to be overweight – and the further six BMI‐underweight respondents who wished to be even thinner than they were. These results suggest that several respondents (2.6% of the total) not yet reporting clinical AN features were both underweight and had a disturbed body perception, an important factor for the onset and progression of AN 47, 48, 49.

Nevertheless, most respondents in this study desired somewhat larger figures. This result fits with a larger body ideal in LMICs and might be protective against AN development 25. Overweight respondents exhibited the greatest CDRS body satisfaction, with some even desiring a larger figure than they currently have. This finding is in line South African National Health and Nutrition Examination Survey data, which found that overweight black South African women were more satisfied with their weight than women from other ethnicities 8, 50.

Thus, our sample neither entirely held to the typical Western body ideal of thinness nor the traditional African body ideal, in which larger body size represents wealth, beauty and the absence of illness 51, while thinner bodies are perceived as ‘unhappy’ and ‘weak’ 26. This intermediate situation might reflect a period of transition for the body ideal of Burkinabé girls. Longitudinal data would be needed to directly test this hypothesis; however, it was notable that respondents most exposed to media were least likely to desire a larger body than they had, although media exposure did not independently predict ED symptoms.

In addition to overlapping ideals of beauty, there appear to be overlapping sources of body dissatisfaction in this sample. While many respondents wished to be larger, possibly reflecting limited ability to access food, a minority were dissatisfied in ways that suggest possible AN‐related tendencies. Future research should disentangle these sources of dissatisfaction to ensure that we avoid both overestimating ED prevalence and overlooking signs of emerging ED. For this work, it would be important to develop materials, including screening questionnaires, which distinguish between AN‐related and AN‐unrelated body dissatisfaction for use in resource‐poor settings.

### Strengths and limitations

Several limitations of the current study need to be considered. First, due to the cross‐sectional study design, we are unable to determine temporal ordering of events and thus causal relationships. This is particularly important given that our data suggest a transition in terms of body image in this setting towards a thinner body ideal, and potentially towards more clinical ED. Testing this transition hypothesis would require follow‐up in the future. Second, all the data provided here were self‐reported to non‐clinical fieldworkers. Despite rigorous training, the fieldworkers may not have delivered the SCID‐5 questions precisely as intended, and thus these findings should be treated as preliminary. The less‐clinical EDE‐Q and CDRS questions may be less susceptible to imperfect delivery error. Third, care must be taken in generalising our findings beyond the study setting. It would seem likely that our results are applicable to other low‐income, low media‐saturation settings in SSA and perhaps elsewhere, but this should be tested empirically rather than assumed. A final shortcoming is that the present study explored complex psychosocial health aspects solely quantitatively with standardised scales, some of which have not been previously validated. Our results provide first, explorative insights on ED in rural West Africa; future research should use locally validated quantitative scales and mixed quantitative‐qualitative approaches, to deepen our understanding of ED in this world region.

## Conclusions

We find that, while clinical ED are rare, several attitudes that predict ED elsewhere are present in rural Burkinabé adolescent females. Such findings highlight the importance of sensitising clinicians to the possibility of ED in SSA, and suggest that AN, BN and BED may increase in West Africa in the future. Our findings that body ideals and self‐image in adolescent Burkinabés appear to be shifting point to the importance of tracking changes in exposure to HIC ideals, and subsequently in attitudes, even in resource‐poorest communities of SSA. If changes in body image accelerate, psycho‐educative body image interventions might help young women in such settings to protect their physical and mental health.

## Supporting information


**Table S1.** Rotated factor loadings for Eating Disorder Examination‐Questionnaire, 7‐item version in female adolescents in rural Burkina Faso.
**Table S2.** Bivariate Poisson regression analyses between common ED predictors and ED symptoms.
**Table S3.** Bivariate linear regression analyses between common ED predictors and ED precursors.
