# Supplementary material for: Eating disorders, body image and media exposure among adolescent girls in rural Burkina Faso
Source: Trop Med Int Health. 2020 Jan 26;25(1):132–41. doi: 10.1111/tmi.13340 (PMC8609432; doi:10.1111/tmi.13340)
Supplement: Supplementary file 1 — Table S1. Rotated factor loadings for Eating Disorder Examination‐Questionnaire, 7‐item version in female adolescents in rural Burkina Faso. Table S2. Bivariate Poisson regression analyses between common ED predictors and ED symptoms. Table S3. Bivariate linear regression analyses between common ED predictors and ED precursors. [file TMI-25-132-s001.docx]

**Supplementary Table 1.** Rotated factor loadings for Eating Disorder Examination-Questionnaire, 7-item version in female adolescents in rural Burkina Faso

| **Scale item** | **Dietary Restraint** | **Shape/Weight  Overvaluation** | **Body Dissatisfaction** |
| --- | --- | --- | --- |
| Restraint over eating | 0.81 | 0.03 | 0.05 |
| Food avoidance | 0.84 | 0.06 | 0.03 |
| Dietary rules | 0.87 | -0.03 | -0.02 |
| Importance of weight | 0.01 | 0.94 | 0.22 |
| Importance of shape | 0.04 | 0.95 | 0.19 |
| Dissatisfaction with weight | 0.04 | 0.19 | 0.95 |
| Dissatisfaction with shape | 0.02 | 0.22 | 0.94 |

**Supplementary Table 2.** Bivariate Poisson regression analyses between common ED predictors and ED symptoms

|  | SCID AN sum score | | SCID BN sum score | | SCID BED sum score | |
| --- | --- | --- | --- | --- | --- | --- |
|  | IRR | 95%CI | IRR | 95%CI | IRR | 95%CI |
| Media exposure | 1.09 | [1.02 – 1.18] | 1.01 | [0.77 – 1.32] | 0.74 | [0.58 – 0.94] |
| Currently not in school vs. in school | 1.07 | [0.85 – 1.37] | 1.79 | [0.77 – 4.19] | 2.82 | [1.39 – 5.72] |
| WHO BMI-for-age z-scores | 1.13 | [1.01 – 1.25] | 1.56 | [1.06 – 2.30] | 1.45 | [1.07 – 1.95] |
| Sexual harassment/violence | 1.25 | [1.13 – 1.39] | 1.58 | [1.16 – 2.15] | 1.36 | [1.05 – 1.77] |
| Age (years) |  |  |  |  |  |  |
| 12-13 | 0.55 | [0.38 – 0.79] | 0.52 | [0.14 – 1.94] | 0.26 | [0.08 – 0.83] |
| 14-15 | 0.94 | [0.67 – 1.30] | 0.59 | [0.16 – 2.20] | 0.44 | [0.16 – 1.22] |
| 16-17 | 1.02 | [0.72 – 1.43] | 1.72 | [0.58 – 5.14] | 1.53 | [0.70 – 3.37] |
| 18-20 | 1 |  | 1 |  | 1 |  |
| CDRS difference score | 0.94 | [0.89 – 0.98] | 1.00 | [0.85 – 1.19] | 1.14 | [1.00 – 1.30] |
| village of residence |  |  |  |  |  |  |
| Nouna | 1.36 | [1.07 – 1.74] | 1.50 | [0.65 – 3.48] | 0.90 | [0.45 – 1.81] |
| EDE-Q |  |  |  |  |  |  |
| dietary restraint | 1.17 | [1.09 – 1.27] | 1.07 | [0.77 – 1.51] | 1.20 | [0.97 – 1.47] |
| shape/weight overvaluation | 1.06 | [1.02 – 1.09] | 1.13 | [1.02 – 1.25] | 1.17 | [1.08 – 1.25] |
| body dissatisfaction | 1.06 | [1.03 – 1.09] | 1.11 | [1.01 – 1.22] | 1.16 | [1.08 – 1.25] |

SCID: Structured Clinical Interview for DSM-5; AN: Anorexia Nervosa; BN: Bulimia Nervosa; BED: Binge Eating Disorder; BMI: Body Mass Index; CDRS: Thompson and Gray’s Contour Drawing Rating Scale; EDE-Q: Eating Disorder Questionnaire; IRR: Incidence Rate Ratio; CI: Confidence Interval

**Supplementary Table 3.** Bivariate linear regression analyses between common ED predictors and ED precursors

|  | CDRS difference score | | EDE-Q  body dissatisfaction factor | |
| --- | --- | --- | --- | --- |
|  | B | 95%CI | B | 95%CI |
| Media exposure | -0.23 | [-0.35 – -0.10] | -0.04 | [-0.22 – 0.14] |
| Currently not in vs in school | -0.20 | [-0.59 – 0.19] | -0.15 | [-0.71 – 0.41] |
| WHO BMI-for-age z-scores | -0.83 | [-0.99 – -0.67] | -0.31 | [-0.56 – -0.07] |
| Sexual harassment/violence | -0.42 | [-0.62 – -0.22] | -0.17 | [-0.46 – 0.13] |
| Age (categorized) | -0.39 | [-0.56 – -0.21] | -0.10 | [-0.34 – 0.15] |
| CDRS difference score | - | - | 0.27 | [0.16 – 0.38] |
| village of residence |  |  |  |  |
| Nouna vs. other residence | 0.89 | [0.49 – 1.29] | 0.51 | [-0.07 – 1.09] |
| EDE-Q |  |  |  |  |
| dietary restraint | -0.47 | [-0.65 – -0.29] | 0.21 | [-0.06 – 0.48] |
| shape/weight overvaluation | -0.01 | [-0.08 – 0.05] | 0.49 | [0.41 – 0.57] |
| body dissatisfaction | 0.13 | [0.08 – 0.18] | - | - |

CDRS: Thompson and Gray’s Contour Drawing Rating Scale; EDE-Q: Eating Disorder Questionnaire; WHO: World Health Organization; BMI: Body Mass Index; B: regression coefficient; CI: Confidence Interval
